# Emergency Medicine Residents Experience Acute Stress While Working in the Emergency Department

**DOI:** 10.5811/westjem.2021.2.51963

**Published:** 2021-04-28

**Authors:** Matthew L. Wong, Leon D. Sanchez, Gregory A. Peters

**Affiliations:** * Beth Israel Deaconess Medical Center, Department of Emergency Medicine, Boston, Massachusetts; † Brigham and Women’s Faulkner Hospital, Department of Emergency Medicine, Boston, Massachusetts; ‡ Massachusetts General Hospital, Department of Emergency Medicine, Boston, Massachusetts

We were very interested to read the manuscript by Janicki and colleagues, and we are grateful for their contribution to the literature.^1^ We agree that stress is a major problem for emergency physicians.^2^ But we had two concerns with the study design.

We performed a similar study with wearable devices and photoplethysmography but had a problem with motion artifact that precluded using all of the collected data in analysis.^3^ Did the authors also experience any problems with motion artifact interfering with data analysis? It is hard to imagine that there were no problems.

We do not think that the authors made a fair comparison when they measured the heart rate and heart rate variability of subjects during didactics and compared them to clinical shift work. We have previously found that our residents walk on average 2.6 miles per shift, 588 steps per hour.^4^ The simple physical activity of clinical shift work should cause some change in heart rate variability, which may account for a lot of the observed difference and not stress.
